# Effect of fish oil on circulating asymmetric dimethylarginine and adiponectin in overweight or obese patients with atrial fibrillation

**DOI:** 10.1002/fsn3.1518

**Published:** 2020-03-11

**Authors:** Elnaz Khorrami, Mohammad Javad Hosseinzadeh‐Attar, Ahmad Esmaillzadeh, Elham Alipoor, Mostafa Hosseini, Zahra Emkanjou, Roya Kolahdouz Mohammadi, Sina Moradmand

**Affiliations:** ^1^ Department of Clinical Nutrition School of Nutritional Sciences and Dietetics Tehran University of Medical Sciences Tehran Iran; ^2^ Cardiac Primary Prevention Research Center (CPPRC) Tehran Heart Center Tehran University of Medical Sciences Tehran Iran; ^3^ Centre of Research Excellence in Translating Nutritional Science to Good Health The University of Adelaide Adelaide Australia; ^4^ Department of Community Nutrition School of Nutritional Sciences and Dietetics Tehran University of Medical Sciences Tehran Iran; ^5^ Department of Nutrition School of Public Health Iran University of Medical Sciences Tehran Iran; ^6^ Department of Epidemiology and Biostatistics School of Public Health Tehran University of Medical Sciences Tehran Iran; ^7^ Department of Cardiology Shahid Rajaei Heart Center Iran University of Medical Sciences Tehran Iran; ^8^ Department of Cardiology Tehran University of Medical Sciences Tehran Iran

**Keywords:** adiponectin, asymmetric dimethylarginine, atrial fibrillation, fish oil, obesity

## Abstract

Obesity and adipose‐derived peptides might be involved in the pathogenesis of atrial fibrillation (AF). Adiponectin plays a major role in the modulation of several metabolic pathways, and asymmetric dimethylarginine (ADMA) has been suggested to be predictive of AF and associated adverse events. The aim of this study was to investigate the effect of fish oil supplementation on circulating adiponectin and ADMA in overweight or obese patients with persistent AF. In this randomized, double‐blind, placebo‐controlled trial, 80 overweight or obese (body mass index (BMI) ≥ 25 kg/m^2^) patients with persistent AF were randomly assigned to two groups to receive either 2 g/day fish oil or placebo, for 8 weeks. Serum levels of adiponectin and ADMA, and anthropometric indexes were measured. This study showed that serum adiponectin concentrations increased significantly following fish oil supplementation compared with the placebo group (13.15 ± 7.33 vs. 11.88 ± 6.94 µg/ml; *p* = .026). A significant reduction was also observed in serum ADMA levels in the fish oil compared with the placebo group following the intervention (0.6 ± 0.13 vs. 0.72 ± 0.15 µmol/L; *p* = .001). The changes in serum adiponectin and ADMA concentrations remained significant after adjustments for baseline values, age, sex, and changes of BMI and waist circumference (*p* = .011 and *p* = .001, respectively). In conclusion,  8 weeks supplementation with fish oil increased serum adiponectin and decreased ADMA concentrations in overweight or obese patients with persistent AF. As adiponectin and ADMA are suggested to be involved in many pathways associated with AF, the current findings might be promising in the clinical management of this disease, an issue that needs further investigations.

## Graphical abstract

This study showed supplementation with 2 g/day fish oil for 8 weeks could increase serum adiponectin and decrease serum ADMA concentrations significantly. Adiponectin is a protective factor associated with many metabolic pathways involved in AF pathogenesis and ADMA has been considered a prognostic factor for AF risk and its related adverse events.

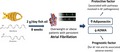

## 1 INTRODUCTION

Atrial fibrillation (AF) is the most common form of arrhythmia, which could increase the risk of stroke and thromboembolic events (Go et al., 2001). It was estimated that 2.3 million people were affected by AF in the United States in 2001, which will increase to more than 5.6 million by the year 2050 (Go et al., 2001). Obesity is considered as an independent risk factor of short‐ and long‐term incidence of AF. Each unit increase in body mass index (BMI) was associated with about 4% increased risk of AF (Tedrow et al., 2010; Wang et al., 2004). Obesity might contribute to AF through mechanisms such as inflammation and oxidative stress, insulin resistance, changes in metabolic by‐products such as advanced glycation end products and related disturbances in myocardial metabolism, which are correlated with atrial structural and functional abnormalities (Abed & Wittert, 2013; Goudis, Korantzopoulos, Ntalas, Kallergis, & Ketikoglou, 2015). Endothelial dysfunction is another obesity‐associated disorder that might contribute to AF (O'Neal et al., 2014).

Other factors such as hypertension and epicardial fat might also contribute to atrial electro‐structural dysfunction (Abed & Wittert, 2013; Goudis et al., 2015). Epicardial fat has strong relationship with BMI and especially with visceral adipose tissue (Rabkin, 2014). Adipose tissue produces lots of bioactive molecules or adipocytokines, which are involved in different physiological and pathophysiological pathways. Adiponectin is a major adipokine with well‐known insulin‐sensitizing, anti‐inflammatory, antiproliferative, and anti‐atherogenic properties (Ouchi, Parker, Lugus, & Walsh, 2011). Lower release of epicardial adiponectin was associated with development of AF after cardiac surgery (Kourliouros et al., 2011). Circulating adiponectin was significantly lower in AF compared to controls with normal sinus rhythm (Choi et al., 2012), and was inversely associated with major cardiovascular events in women with AF (Hernández‐Romero et al., 2013). Asymmetric dimethylarginine (ADMA), a major inhibitor of nitric oxide (NO) synthase, is another peptide secreted by adipocytes (Spoto, Parlongo, Parlongo, Sgro, & Zoccali, 2007), which can induce endothelial dysfunction, inflammation, and oxidative stress in cardiovascular disorders (Bouras et al., 2013). ADMA levels were significantly higher in AF compared to non‐AF individuals candidate for coronary angiography (Chao et al., 2013) and healthy controls (Cengel et al., 2008). Higher ADMA levels were associated with increased risk of AF recurrence following electrical cardioversion (Xia et al., 2008) and catheter ablation (Yang et al., 2011). It has been suggested that ADMA could have prognostic value in predicting AF in high‐risk patients (Stamboul et al., 2015). Besides, studies have indicated the predictive value of ADMA in determining the adverse events such as mortality and stroke in AF (Chao et al., 2013; Horowitz et al., 2018).

Little is known about the effects of nutritional supplements on circulating levels of these biomarkers in patients with AF. Higher serum levels of long‐chain omega‐3 fatty acids have been linked with a lower incidence of AF (Virtanen, Mursu, Voutilainen, & Tuomainen, 2009). Moreover, fish oil supplementation led to lower incidence of AF (Calò et al., 2005), duration of AF and ICU stay (Farahani et al., 2017) after coronary artery bypass graft surgery (CABG).

However, studies investigating the effect of fish oil supplementation on these mediating factors are scarce in patients with AF. Thus, the aim of the current study was to examine the effect of fish oil supplementation on serum ADMA and adiponectin concentrations in overweight or obese patients with persistent AF.

## 2 MATERIALS AND METHODS

### 2.1 Study population

In the current randomized, double‐blind, placebo‐controlled, parallel design trial, 80 overweight or obese (25 ≤ BMI ≤ 40 kg/m^2^) patients with persistent AF, age ≥ 50 years, and on warfarin therapy were enrolled.

Patients with thyroid dysfunction, valvular heart disease, heart failure, and CABG in the past 3 months were not included. Besides, smokers, patients with a history of drug or alcohol abuse, those with recent acute illnesses and infectious diseases, woman with hormone replacement therapy, participants consuming fish oil derivatives in the past 3 months, or allergic to fish oil were not included in this study. Patients were excluded if they need CABG or did not consume more than 10% of capsules. The sample size was calculated, considering adiponectin as the main variable, based on the formula for randomized clinical trials, with type I (a) error of 0.05 and type II error (b) of 0.20 (power = 80%). The calculation showed that 38 participants in each group would be sufficient. Assuming a dropout of 10%, the final sample size was determined to be 42 patients in each group. Ethical Committee of Tehran University of Medical Sciences approved the study protocol, and informed written consent was obtained from all patients. This clinical trial was registered with a national registry system of clinical trials (Registration ID at irct.ir: IRCT2012110411362N1).

### 2.2 Study design

Patients were assigned to two groups using random stratified permuted blocks. Two diabetes categories (yes/no) were considered as strata to eliminate the confounding effect of this disease. Patients were randomly assigned to two groups to receive either 2 g/day (two 1,000‐mg capsules) fish oil (each capsule contained 300 mg eicosapentaenoic acid (EPA) and 200 mg docosahexaenoic acid (DHA)) or 2 g/day olive oil as placebo for 8 weeks. Fish oil supplements and placebo were produced by Daana Company. The placebo and fish oil capsules were the same in size, weight, and color.

Patients were followed up by phone weekly. To assess compliance, we asked the participants to bring back the empty box of the capsules at the end of the study. All patients were requested not to change their usual physical activity and dietary intakes throughout the study period and not to consume any additional supplement. A 3‐day 24‐hr dietary recall (two weekdays and one weekend) was completed to examine reported dietary intakes at baseline and following the intervention. Mean dietary intakes were analyzed using Nutritionist IV software (N Squared Computing) modified for some local foods.

### 2.3 Assessment of variables

Anthropometric indices including height and weight were measured using Seca scale and tape (Germany) before and after the intervention, with light clothing and no shoes. BMI was calculated as weight in kilograms divided by height in meters squared. Waist circumference was measured between the lowest rib and the iliac crest, using a nonelastic tape. An overnight 10‐ml fasting venous blood sample was taken at baseline and at the end of the trial. Samples were centrifuged at 3,000 rpm for 15 min to separate serum and stored at −80°C until the analysis. Commercial enzyme‐linked immunosorbent assay (ELISA) kits were used to quantify serum levels of adiponectin (Mediagnost) and ADMA (DLD). Intra‐ and interassay coefficients of variation were <10% for these measurements.

### 2.4 Statistical methods

Normal distribution of the variables was assessed using the Kolmogorov–Smirnov test. Independent samples *t* test or Mann–Whitney *U* test was applied to compare variables between the two groups at baseline. Chi‐square test was used to compare categorical variables between the two groups. To examine the effect of supplementation on main variables, we applied analysis of covariance (ANCOVA) adjusting for baseline measurements as well as, age, sex, and changes in BMI and waist circumference. *p* values were considered significant at the level of ˂.05. Statistical analysis was performed using SPSS software (IBM SPSS Statistics for Windows, Version 18).

## 3 RESULTS

### 3.1 General characteristics

**Figure 1:**
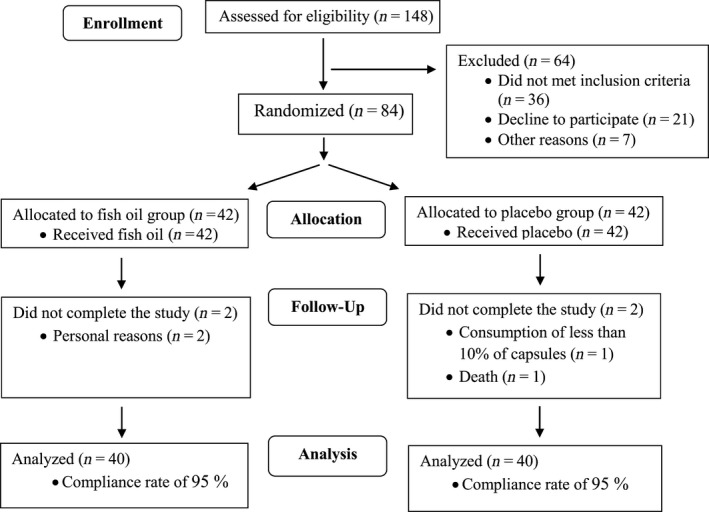
CONSORT flow diagram of the study

Four patients did not finish the study: two in the fish oil group due to personal reasons and two in the control group due to consuming <10% of the capsules (*n* = 1) and death (*n* = 1) (Figure 1). No adverse effects were reported by patients after taking supplements or placebos.

General baseline characteristics of the study participants are shown in Table 1. No significant differences were observed in mean age, BMI, and waist circumference between the fish oil and placebo groups at baseline. Moreover, the prevalence of diabetes, hypertension, and hypercholesterolemia was not different between the two groups. Reported dietary intakes of energy, macronutrients, EPA, DHA, and α‐linolenic acid (ALA) were not significantly different between the two groups at baseline (Table 1). Besides, there were no considerable differences in reported dietary intakes at the end of the study.

**Table 1:** Baseline characteristics of the study participants

	Group		*p*
	Control (*n* = 40)	Fish oil (*n* = 40)	
Age, years (Mean ± *SD*)	69.7 ± 7.7	68.6 ± 8.3	.523*
Male, *n* (%)	19 (47.5)	18 (45)	>.9**
Diabetes, *n* (%)	11 (27.5)	11 (27.5)	>.9**
Metformin, *n* (%)	9 (22.5)	9 (22.5)	>.9**
Hypercholesterolemia, *n* (%)	22 (55)	22 (55)	>.9**
Atorvastatin, *n* (%)	19 (47.5)	22 (55)	.655**
Dietary intake (Mean ± *SD*)			
Energy (kcal/day)	1,453.8 ± 543.7	1,319.8 ± 443.1	.248***
Protein (g/day)	57.2 ± 26.9	48.8 ± 22.3	.148***
Fat (g/day)	40.7 ± 21.3	36.5 ± 15.9	.641***
Carbohydrate (g/day)	220.6 ± 88.8	189.6 ± 80.1	.117***
EPA (mg/day)	0.02 ± 0.7	0.02 ± 0.7	.542***
DHA (mg/day)	0.7 ± 0.2	0.7 ± 0.2	.580***
ALA (mg/day)	0.22 ± 0.2	0.24 ± 0.2	.791***

Note

Data are presented as mean ± *SD* or frequency (%).

* Based on independent samples *t* test.

** Based on chi‐square test.

*** Based on Mann–Whitney test.

### 3.2 ADMA and adiponectin

Serum adiponectin concentrations increased significantly following fish oil supplementation compared with the control group (13.15 ± 7.33 vs. 11.88 ± 6.94 µg/ml, *p* = .026). Furthermore, a significant reduction was observed in serum ADMA levels in the fish oil compared with the control group following the intervention (0.6 ± 0.13 vs. 0.72 ± 0.15 µmol/L, *p* = .001). Changes in serum adiponectin and ADMA remained significant after adjustment for baseline values, age, sex, changes of BMI and waist circumference (*p* = .011 and *p* = .001, respectively) (Table 2).

**Table 2:** Changes in adiponectin and ADMA concentrations in patients with AF before and following 8‐week supplementation with fish oil

	Control (*n* = 40)	Fish oil (*n* = 40)	Diff	95% CI		*p*
	Mean ± *SD*	Mean ± *SD*		Lower	Upper	
Adiponectin (µg/ml)						
Before	12.38 ± 7	12.34 ± 6.8	0.04	−3.032	3.111	.979*
After	11.88 ± 6.94	13.15 ± 7.33	−1.309	−2.453	−0.164	.026**
Change	0.51 ± 2.37	−0.8 ± 2.74	1.31	0.168	2.452	.025*
*p* ***	0.184	0.072				
ADMA (µmol/L)						
Before	0.69 ± 0.13	0.64 ± 0.13	0.049	−0.008	0.106	.088*
After	0.72 ± 0.15	0.6 ± 0.13	0.104	0.043	0.164	.001**
Change	−0.03 ± 0.17	0.05 ± 0.14	−0.072	−0.142	−0.004	.04*
*p* ***	0.339	0.04				

Note

*p* values in the rows refer to pre‐ to post‐differences within the each study group.

* Based on independent samples *t* test.

** Adjusted for baseline values based on ANCOVA.

*** Based on paired samples test.

### 3.3 Anthropometric changes

Weight (0.99 ± 1.97 vs. 0.06 ± 1.74 kg, *p* = .029), BMI (0.40 ± 0.84 vs. 0.03 ± 0.7 kg/m^2^, *p* = .029), and waist circumference (2.42 ± 2. 15 vs. 0.1 ± 1.34 cm, *p* < .001) were significantly decreased in the fish oil compared to the control group (Table 3).

**Table 3:** Changes in anthropometric indexes in patients with AF before and following 8‐week supplementation with fish oil

	Control (*n* = 40)	Fish oil (*n* = 40)	Diff	95% CI		*p*
	Mean ± *SD*	Mean ± *SD*		Lower	Upper	
Weight (kg)						
Before	73.8 ± 12.9	73.6 ± 13.3	0.23	−5.604	6.064	.938*
After	73.7 ± 13	72.6 ± 13.3	0.927	0.095	1.758	.029**
Change	0.06 ± 1.74	0.99 ± 1.97	−0.925	−1.751	−0.099	.029*
*p* ***	0.821	0.003				
Body mass index (kg/m^2^)						
Before	29.2 ± 4.2	29 ± 4.9	0.164	−1.887	2.215	.874*
After	29.2 ± 4.3	28.6 ± 4.8	0.382	0.04	0.725	.029**
Change	0.03 ± 0.7	0.4 ± 0.84	−0.378	−0.722	−0.034	.032*
*p* ***	0.826	0.004				
Waist circumference (cm)						
Before	100.3 ± 8.9	102.3 ± 16	−1.963	−7.715	3.79	.499*
After	100.2 ± 8.8	99.9 ± 16.2	2.321	1.517	3.126	<.001**
Change	0.1 ± 1.34	2.42 ± 2.15	−2.32	−3.119	−1.521	<.001*
*p* ***	0.640	<0.001				

Note

*p* values in the rows refer to pre‐ to post‐differences within the each study group.

* Based on independent samples on *t* test.

** Adjusted for the baseline values based on ANCOVA.

*** Based on paired samples test.

## 4 DISCUSSION

In this randomized, placebo‐controlled, parallel design clinical trial, 2 g/daily fish oil supplementation for 8 weeks had a significant effect on increasing adiponectin and decreasing ADMA serum concentrations.

The effect of n‐3 polyunsaturated fatty acids on increasing circulating adiponectin has been well recognized in experimental and clinical models (Flachs et al., 2006; Itoh et al., 2007). However, little is known about the effect of these fatty acids on adiponectin in AF. EPA treatment (300 mg/kg daily for 4 weeks) in a rabbit model declined the increased duration of AF and atrial fibrosis associated with heart failure. This change was accompanied with increased adiponectin and decreased tumor necrosis factor alpha (TNF‐α) expression in the atrium and epicardial adipose tissue (Kitamura et al., 2011). A clinical study showed neither a difference in the incidence of postcardiac surgery AF nor a change in adiponectin concentration compared to controls following 900 mg daily consumption of EPA for 3–6 months (Yamamoto et al., 2014).

Left ventricular (LV) hypertrophy is a risk factor for AF development (Healey & Connolly, 2003). In an animal study of LV hypertrophy, adiponectin levels increased significantly in both normal low‐fat and high‐fat diets supplemented with fish oil (2.3% of energy intake as EPA + DHA). Besides, a normal low‐fat diet supplemented with EPA + DHA prevented LV hypertrophy (Shah et al., 2009). Another experimental study showed that fish oil‐enriched diet (5%) prevented LV hypertrophy, increased serum adiponectin concentration, and suppressed inflammatory markers compared to corn oil diet (5%) (Halade, Williams, Lindsey, & Fernandes, 2011). A large population‐based study showed that low adiponectin levels could be associated with higher marker of LV hypertrophy (Pääkkö, Ukkola, Ikäheimo, & Kesäniemi, 2010).

Adiponectin has been proposed as a marker of peroxisome proliferator‐activated receptor‐gamma (PPAR‐γ) activity (Wagner et al., 2009) and also a mediator of multiple effects of PPAR‐γ (Bouskila, Pajvani, & Scherer, 2005) and PPAR‐α (Tsuchida et al., 2005). PPAR‐γ mRNA was decreased significantly in AF compared to non‐AF individuals, and also in persistent AF compared to the paroxysmal type (Chen et al., 2009). PPAR‐γ agonists, such as pioglitazone, reduce atrial remodeling and AF duration significantly, potentially through antioxidant, anti‐inflammatory, and anti‐apoptotic mechanisms (Liu, Liao, Yang, & Tang, 2016; Xu et al., 2012). Omega‐3 fatty acids are natural agonists of PPAR‐γ and PPAR‐α (Grygiel‐Górniak, 2014).

Adiponectin could also inhibit angiotensin II‐induced cardiac inflammation and fibrosis by increasing macrophage autophagy and up‐regulation of anti‐inflammatory mediators (Fujita et al., 2008; Qi, Jia, Li, Li, & Du, 2014). Low circulatory levels of adiponectin contribute to insulin resistance and endothelial vascular dysfunction (Goldstein & Scalia, 2004), which could be involved in AF pathogenesis (Abed & Wittert, 2013; Goudis et al., 2015; O'Neal et al., 2014).

Accordingly, these studies suggest that despite some observational studies indicating a direct relationship between adiponectin and higher risk of AF in a population‐based cohort (Macheret et al., 2015) and after catheter ablation in patients with paroxysmal AF (Kim et al., 2017), this peptide may modulate pathophysiological pathways in AF. Higher adiponectin levels in those studies might play a compensatory role to control AF.

In the current study, ADMA serum concentration decreased in patients with persistent AF following 2 g/day fish oil supplementation for 8 weeks.

It has been reported that serum ADMA levels decreased following 1,800 mg daily EPA treatment in patients in chronic phase of cerebral infarction (Hagiwara, Nishiyama, & Katayama, 2011). However, 1‐month supplementation with 1 g/day n‐3 PUFAs in patients with acute myocardial infarction (Haberka et al., 2011) or 2.4 g/day n‐3 PUFAs for 36 months in men with hypercholesterolemia (Eid et al., 2006) did not affect ADMA levels.

However, these studies had different designs and pathophysiological backgrounds and little is known about the effect of n‐3 fatty acids on ADMA concentration in AF.

Asymmetric dimethylarginine has been proposed to have prognostic value in determining AF in high‐risk patients (Stamboul et al., 2015) and the adverse events such as mortality and stroke in AF (Chao et al., 2013; Horowitz et al., 2018). Treatment with 10 mg daily rosuvastatin for 3 months following elective electrical cardioversion decreased recurrence of AF through reduction of ADMA levels (Xia, Yin, Li, Song, & Qu, 2009). ADMA might be involved in atrial remodeling. Higher ADMA levels were correlated with high incidence of non‐pulmonary veins ectopies, which were predictive of AF (Yang et al., 2011). Increased ADMA concentration in AF could be associated with NO deficiency, which in turn leads to cardiomyocyte apoptosis (Feng et al., 2002). ADMA has been suggested as a link between AF with oxidative stress and inflammation, endothelial dysfunction, and atherosclerosis (Chao et al., 2013). Inflammation is involved in AF pathogenesis (Abed & Wittert, 2013; Goudis et al., 2015). ADMA is considered a determinant and biochemical measure of endothelial dysfunction (Paiva et al., 2010), which might also contribute to AF (O'Neal et al., 2014).

Adiponectin and ADMA are adipose‐derived factors. Thus, it might be speculated that their changes in this study originated from decrease in anthropometric indexes not fish oil supplementation. However, it should be considered that changes in weight and BMI, although statistically significant, are negligible and clinically irrelevant. The potential effect of BMI and waist circumference changes on adiponectin and ADMA was also considered in statistical analysis. Additionally, considering the BMI cutoff of more than 25 kg/m^2^ confined the effect of weight status on adipokines’ changes and was a strength of this study as many patients with AF are overweight or obese.

Some limitations are needed to be mentioned for this trial. Our study provided observations on changes of adiponectin and ADMA serum concentrations following fish oil supplementation in patients with persistent AF, but did not directly evaluate clinical outcomes and mechanisms associated with adiponectin and ADMA. However, considering the important and close relationship of these factors with mechanisms involved in AF pathogenesis and the predictive and prognostic role of ADMA in AF, the corresponding changes following fish oil supplementation are considerable and should be investigated with more details in future studies.

## 5 CONCLUSION

Eight weeks of fish oil supplementation led to increase in serum adiponectin and decrease in serum ADMA concentrations in patients with persistent AF.

## CONFLICT OF INTEREST

The authors declare that they do not have any conflict of interest.

## ETHICAL APPROVAL

This study was approved by the Institutional Review Board of Tehran University of Medical Sciences.

## INFORMED CONSENT

Written informed consent was obtained from all study participants.
